# The Causes of Elephantiasis—A New Tinea

**Published:** 1878-10

**Authors:** 


THE CAUSES OF ELEPHATIASIS—A NEW TINEA.—We are supposed to prevent the coolie trade in American bottoms and patrol this part of the coast of China. Consequently we oscillate between this port and Swatow. We have not yet seen anything to prevent, and swelter in heat and discomfort for nothing. But, being at Ammoy so much, time has made me acquainted with Dr. Patrick Manson, the physician of the community. He, in common with Dr. Lewis, of India, and Dr. Bancroft, of Austria, has discovered and made known the existence of the filaria sanguinis hominis. Lewis and Bancroft knew of the parasite perhaps a few months before Manson did, but the latter not only discovered it in the blood of numerous patients, but further investigated and labored, and finally made known the complete life of the parasite. He discovered that the common mosquito is the intermediary host of the filaria sanguinis hominis. This parasite lives, or has its habitat, in the lymphatic vessels, its presence eventually disordering the functions and preventing the flow of the fluid; hence fulness of the distal vessels, hypertrophy and formation of the elephantoid tissue, a low-grade material, lymph scrotum, chyluria, etc., are effects due to it. I am quite convinced of the truth of these observations of Dr. Manson. I have seen young filaria time and time again in the blood of patients in the Chinese hospital here, and have noted any number of elephantoid and similar cases in connection, and thoroughly believe in it as cause and effect.

By this discovery elephantiasis is preventable. Where the mosquito flourishes most, and where there exists one case, any number of cases may occur. The mosquito gets the young filaria from man. It undergoes one stage of its life, or metamorphosis, in the mosquito's stomach. It is discharged with the young on water, and in that medium, or by it, infection occurs. Without the mosquito the filaria sanguinis hominis could not be matured. The adult worm causes the mischief, such as elephantoid disease, chyluria, and lymph scrotum. Innumerable young in the blood cause a condition similar to ague, and which is treated similarly. The adult female worm only has been seen.

Dr. Manson has operated upon elephantiasis scroti ninety odd times, with two fatal cases, the tumors being of all sizes and consistence. He operates skilfully, first cutting down and isolating the testes, then clearing the penis, then taking away the mass with the catlin, dissecting up enough skin before doing so to cover the testes. A large sponge is pressed upon the bleeding surface, and the vessels are taken up and ligatured carefully. A drainage-tube in the form of an inverted U is put in, and catgut sutures are employed. Cotton saturated with carbolated oil is the dressing.

Enclosed I send you a pamphlet account of filaria immites—the parasite inhabiting the heart of dogs in this country. It lives in the heart, and disturbs the action of the organ, and sometimes plugs up the pulmonary vessels. The comparative anatomy of the parasite is fully described in the pamphlet, but the full life of it is still unknown, and Dr. Manson is engaged in seeking it.

I would like to present the pamphlet to the Academy of Natural Sciences of Philadelphia, if you will be kind enough to do it. Dr. Leidy would possibly be interested in the matter. Dr. Manson has taken up many insects in hope of finding the intermediary host, but has not yet succeeded.

In the same pamphlet will be found some account of the original investigations and beginnings of Dr. Manson's work on the filaria sanguinis hominis; also an account of the filaria sanguinolenta, another parasite affecting the dog, but other organs and other tissues than the heart.

I consider these discoveries to be of vast interest and importance. The London *Lancet* has given some account of Dr. Manson's efforts from time to time. Dr. Cobbold knew the results from him.

A new variety of tinea is also being distinguished by

Dr. Manson. It differs from the T. circinata in every particular, clinically and pathologically. The case is from the Straits settlements, and has been known as a ringworm, the local name given it where it occurs, Burmese ringworm, etc. It affects the skin and produces a condition similar to watered silk, one ring within another, and no part healing as the growth progresses. The epidermis is raised up in flakes, rises, and is detached in larger patchcs than in T. circinata. Microscopically, the difference consists in there being few spores, much large-sized and long-pointed mycelium. The whole body becomes gradually affected, no part healing as in cercinata. Dr. Tilbury Fox, of London, is to be written to in regard to it, and will present the cases and notes for Dr. Manson.

Leprosy is common in this part of China, and thus far no remedy, in the true sense of the word, is known here. It is not considered contagious, and no measures of precaution are taken beyond avoidance of contact. Marriage of lepers is not prevented; and women affected not unfrequently beguile men in the common notion of transfering it.

I hope you may get some notion from this of the filaria parasite and the important work done by a physician in busy practice with limited means and poor instruments of research.—*Philadelphia Medical Times.*

